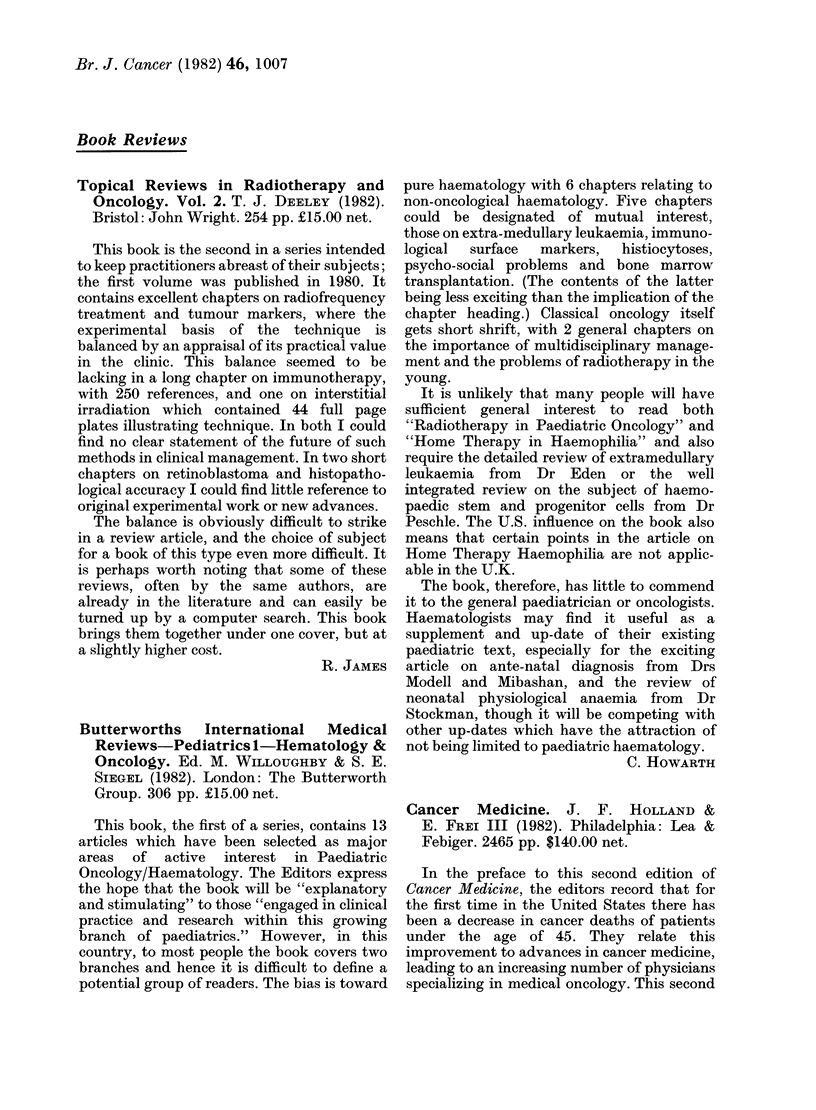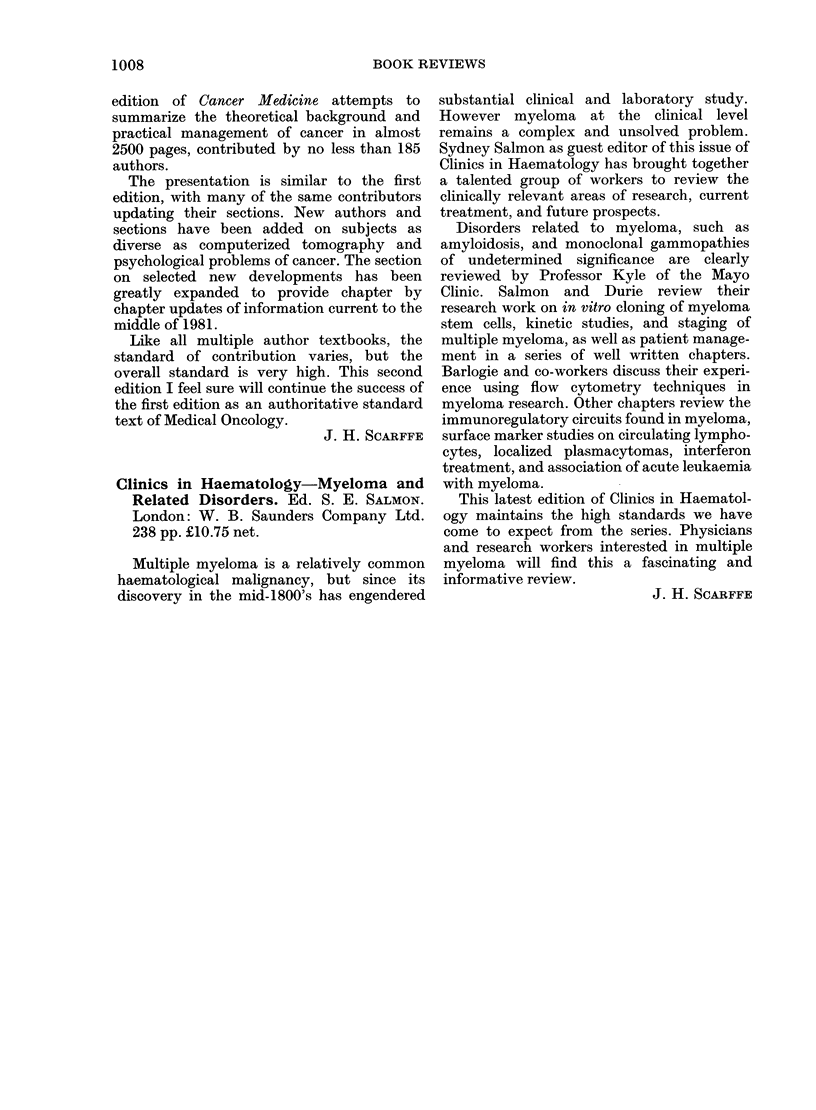# Cancer Medicine

**Published:** 1982-12

**Authors:** J. H. Scarffe


**Cancer Medicine.** J. F. HOLLAND & E. FREI III (1982). Philadelphia: Lea & Febiger. 2465 pp. $140.00 net.

In the preface to this second edition of *Cancer Medicine*, the editors record that for the first time in the United States there has been a decrease in cancer deaths of patients under the age of 45. They relate this improvement to advances in cancer medicine, leading to an increasing number of physicians specializing in medical oncology. This second edition of *Cancer Medicine* attempts to summarize the theoretical background and practical management of cancer in almost 2500 pages, contributed by no less than 185 authors.

The presentation is similar to the first edition, with many of the same contributors updating their sections. New authors and sections have been added on subjects as diverse as computerized tomography and psychological problems of cancer. The section on selected new developments has been greatly expanded to provide chapter by chapter updates of information current to the middle of 1981.

Like all multiple author textbooks, the standard of contribution varies, but the overall standard is very high. This second edition I feel sure will continue the success of the first edition as an authoritative standard text of Medical Oncology.

J. H. Scarffe